# Beyond Moisture Content: Time-Domain NMR for Quantifying Molecular Mobility, Water Accessibility and Food Function

**DOI:** 10.3390/molecules31193476

**Published:** 2026-09-29

**Authors:** Zeev Wiesman

**Affiliations:** Phyto-Lipid Biotechnology Laboratory (PLBL), Department of Biotechnology Engineering, Faculty of Engineering Sciences, Ben-Gurion University of the Negev, Beer-Sheva 84105, Israel; zeev.wiesman@gmail.com

**Keywords:** time-domain NMR, low-field NMR, water mobility, water accessibility, self-diffusion, T_1_, T_2_, food structure, food quality, molecular dynamics

## Abstract

Moisture content is among the most routinely measured properties of foods, yet water quantity alone does not determine processing behavior, stability, texture, fluid release or sensory function. In heterogeneous food matrices, water occupies environments differing in molecular restriction, compartmentalization, exchange, connectivity and translational mobility. Time-domain nuclear magnetic resonance (TD-NMR) provides a rapid and non-destructive means of interrogating these properties through proton signal intensity, longitudinal and transverse relaxation, relaxation-time distributions, self-diffusion and multidimensional correlation measurements. This review develops a framework extending food-water analysis from quantity to molecular state, mobility, translational accessibility and function. Particular emphasis is placed on distinguishing local molecular environments reflected by relaxation from longer-range molecular displacement characterized by diffusion. Applications in meat, plant-based foods, dairy systems, cereals, gels, frozen and dried foods, and edible oils illustrate how TD-NMR descriptors can be connected with water holding, texture, juiciness, processing behavior, oxidation and shelf life. Integration with structural imaging, mechanical measurements, chemometrics and machine learning further positions TD-NMR as a molecular-state sensing platform for predictive food analysis, product design and intelligent manufacturing.

## 1. Introduction: Beyond How Much Water Is Present

Water is one of the dominant constituents of most foods and a major determinant of processing behavior, stability, texture, and sensory performance. Moisture content is therefore routinely measured during formulation, manufacturing, storage, and quality assessment. However, conventional moisture analysis essentially answers only one question: how much water is present?

For many food functions, this information is insufficient. Foods containing similar amounts of water can exhibit markedly different mechanical properties, water-holding capacity, cooking losses, drying kinetics, fluid release, and sensory juiciness. Conversely, foods retaining relatively large amounts of water may exhibit limited fluid release or a dry oral perception when that water is strongly restricted, compartmentalized, or poorly connected within the food architecture. Thus, the functional behavior of water depends not only on its quantity, but also on its molecular environment, mobility, spatial organization, and accessibility to transport.

Time-domain nuclear magnetic resonance (TD-NMR), commonly implemented using low-field permanent-magnet instruments, is particularly well suited to interrogating these properties. TD-NMR has evolved from an established tool for rapid compositional analysis into a versatile analytical platform capable of probing relaxation behavior, molecular mobility, self-diffusion, heterogeneity, and compartmentalization in intact food matrices [1,2,3,4,5]. Recent applications extend from packaged-food assessment [5] and meat water-holding capacity [6] to plant-based food structure and texture [7,8,9], machine-learning-assisted prediction of frozen-food quality [10], and characterization of edible-oil oxidation using diffusion measurements and artificial intelligence [11,12].

Several previous reviews have documented the broad utility and continuing development of TD-NMR for food composition, quality assessment, molecular mobility, imaging, and emerging sensing applications [1,13,14,15]. The objective of the present review is therefore not to provide another exhaustive catalogue of TD-NMR applications in foods. Instead, it develops a mechanistically organized framework for interpreting TD-NMR measurements according to the distinct physical information they provide and for relating these measurements to food structure, transport, and function.

The proposed hierarchy is: water quantity → molecular state → local mobility → compartmentalization/connectivity → translational accessibility → transport/release → food function.

A central premise of this framework is that relaxation and diffusion interrogate related but non-equivalent aspects of molecular behavior. Longitudinal (T_1_) and transverse (T_2_) relaxation primarily report on molecular environments and relaxation dynamics arising from water–matrix interactions, molecular motion, and exchange. By contrast, the self-diffusion coefficient (D) quantifies translational displacement over the experimental observation scale and can therefore provide complementary information about the ability of molecules to move through structurally constrained and interconnected domains. Neither relaxation times nor D should, however, be interpreted as direct measurements of macroscopic fluid transport or release; these processes additionally depend on architecture, connectivity, deformation, pressure gradients, and relevant length and time scales.

Accordingly, this review distinguishes water quantity from relaxation-defined molecular state and local mobility, and further distinguishes these properties from translational accessibility probed by self-diffusion. This separation provides a basis for integrating TD-NMR with structural and mechanical measurements and, increasingly, with multivariate and predictive approaches. It also avoids the common conceptual simplification that moisture content, relaxation behavior, water retention, molecular diffusion, and macroscopic fluid release represent interchangeable measures of the same underlying property.

Scope and literature selection. This article is a narrative, mechanism-oriented review rather than a systematic review or meta-analysis. Literature was selected to represent foundational TD-NMR methodology, major food-system applications, recent developments, and studies relevant to the proposed quantity–state–accessibility–function framework. During revision, coverage from independent research groups was expanded across meat, plant-based foods, dairy systems, gels and emulsions, cereals, freezing, drying, packaged foods, and lipid-rich foods. The selection is illustrative rather than exhaustive, and no formal meta-analytic weighting was applied. The primary focus is water in hydrated food matrices. A limited number of non-aqueous examples are retained to demonstrate that TD-NMR principles also extend to other proton-rich molecular phases; these examples are illustrative extensions and are not included within the definition of water accessibility.

Viewed in this way, TD-NMR can contribute more than compositional or empirical quality measurements. It can provide quantitative descriptors of the molecular state and translational behavior of water within food architecture, helping to bridge the gap between how much water a food contains and how that water participates in processing, stability, texture, transport, and sensory function.

## 2. From Water Quantity to Water Function

Gravimetric moisture determination provides indispensable information for composition, formulation, mass balance and regulatory control. Proton signal amplitude can likewise be calibrated for rapid determination of water or lipid content by TD-NMR [1,2,3]. Neither measurement, however, directly describes how water is organized within a matrix.

Water activity (a_w_) reflects thermodynamic availability and is fundamental to microbial stability and many chemical reactions. Water activity and molecular mobility are related but distinct properties; TD-NMR should therefore not be regarded as an alternative measurement of a_w_.

Water-holding capacity, centrifugation loss, drip loss, pressing loss and cooking loss characterize how effectively a matrix retains water under a defined perturbation. These measurements are particularly important in meat and other protein foods, where structural and biochemical changes alter both water distribution and water retention [16,17,18,19]. However, retention does not directly quantify how readily retained water can redistribute through an intact structural network. This distinction separates three non-equivalent properties: water quantity → water retention → water transport accessibility.

## 3. TD-NMR as a Molecular-State Probe

TD-NMR detects the response of nuclear spins, most commonly ^1^H nuclei, following radiofrequency excitation in a magnetic field. In foods, the dominant mobile proton-containing phases are usually water and lipids. Unlike high-resolution NMR spectroscopy, TD-NMR generally does not seek detailed chemical-shift resolution. Instead, molecular information is obtained from relaxation, signal amplitude, diffusion and related spin-dynamic measurements.

This apparent reduction in chemical detail offers considerable advantages for heterogeneous foods. Measurements can often be performed non-destructively, with little or no sample preparation, repeatedly on the same specimen, using comparatively compact instruments, and on intact multiphase food materials [1,2,3,4,5].

## 4. T_1_ and T_2_: What Relaxation Measures

The longitudinal relaxation time, T_1_, describes recovery of longitudinal magnetization following perturbation and is sensitive to molecular motion, magnetic-field strength, viscosity, temperature and interactions with the surrounding environment. T_1_ should be treated as an experimental molecular descriptor rather than an immutable label for a chemically or thermodynamically defined water fraction.

Transverse relaxation, T_2_, is particularly widely used in food science. Carr–Purcell–Meiboom–Gill measurements produce signal decays that may be represented as discrete exponentials or continuous relaxation-time distributions. Within a defined system and measurement protocol, shorter T_2_ values generally reflect more efficient relaxation and stronger molecular restriction, whereas longer values are frequently associated with comparatively greater molecular mobility [1,4].

The common terminology of “bound”, “immobilized” and “free” water should be applied cautiously. Food water does not generally exist as three universally defined molecular species. Measured relaxation populations are affected by water–macromolecule interactions, compartment size, exchange between compartments, proton exchange, interfacial effects, lipid contributions, temperature and pulse-sequence conditions. Accordingly, the preferred terminology in this review is short-T_2_, intermediate-T_2_ and long-T_2_ relaxation populations/environments, or more generally relaxation-defined molecular environments. More specific assignments should be made only when supported by independent structural, compositional or physicochemical evidence. Terms such as “bound”, “immobilized” and “free” are retained only when describing terminology used in previous literature or when explicitly defined for a particular system.

## 5. Relaxation Distributions as Molecular Fingerprints

Inverse Laplace transformation allows multiexponential relaxation decays to be represented as distributions rather than being constrained to a small number of predetermined exponential components [20]. Relaxation distributions provide descriptors of heterogeneity including peak position, peak width, relative signal contribution, redistribution among populations and appearance or disappearance of molecular environments.

However, inverse Laplace transformation is mathematically ill-conditioned. Signal-to-noise ratio, regularization and inversion parameters can alter the apparent width or number of reconstructed populations [20]. The major analytical value of a relaxation distribution is therefore not necessarily to assign every peak to a rigid water category, but to detect reproducible changes in the distribution of molecular environments.

For reproducibility, studies using relaxation-time distributions should report at minimum the inversion algorithm, regularization approach and parameter-selection procedure, signal-to-noise information, acquisition sampling, the rule used to identify or count peaks, and an estimate of repeatability or uncertainty. Apparent changes in peak number or width should not be interpreted structurally unless they are robust to reasonable variation in inversion settings and supported by replicate measurements.

## 6. Molecular Mobility Is Not Translational Accessibility

The distinction between local molecular dynamics and translational displacement is central to the interpretation of food TD-NMR. T_2_ reflects the relaxation environment experienced by a proton population. A water molecule can therefore exhibit relatively high local mobility while remaining spatially confined within a closed compartment.

Food matrices contain multiple barriers to translational displacement, including protein networks, polysaccharide gels, membranes, lipid interfaces, cell walls, small pores, disconnected cavities, dead-end channels and tortuous pathways. Two matrices may consequently exhibit partially overlapping relaxation behavior while imposing very different restrictions on translational motion. Relaxation asks what molecular environment the proton experiences; diffusion asks how readily the molecule can displace through that environment.

## 7. Self-Diffusion: Quantifying Translational Molecular Motion

Pulsed-field-gradient NMR enables molecular self-diffusion to be quantified by encoding molecular position and measuring signal attenuation associated with displacement [21,22]. For unrestricted diffusion, signal attenuation can be described using the Stejskal–Tanner relationship [21]:

I/I_0_ = exp[−D γ^2^ g^2^ δ^2^ (Δ − δ/3)]

where I/I_0_ is the normalized echo attenuation, γ is the gyromagnetic ratio, g is gradient strength, δ is gradient duration, Δ is the diffusion interval, and D is the self-diffusion coefficient.

In foods, the measured coefficient should frequently be interpreted as an apparent self-diffusion coefficient, D_app_, because displacement may be influenced by geometric restriction, compartment dimensions, tortuosity, interfaces, exchange and diffusion observation time. At minimum, quantitative diffusion studies should report gradient duration (δ), diffusion interval (Δ), gradient range and calibration, pulse sequence, sample temperature and fitting model. When D_app_ is interpreted mechanistically in terms of structural restriction, connectivity or accessibility, measurements at multiple diffusion times are strongly recommended because diffusion-time dependence can help distinguish comparatively unrestricted motion from restriction at relevant structural length scales. A single D_app_ value remains useful for controlled comparative studies, but cross-study comparison is limited when the observation window and acquisition conditions differ. Water-diffusion measurements have, for example, been used to probe meat microstructure [23].

## 8. Water Accessibility as an Operational Descriptor

For the present framework, water accessibility is defined operationally as a scale-dependent physical property: the capacity of water molecules within a food to undergo translational redistribution through the structural environment over a defined spatial and temporal observation scale. Accessibility is not a directly fitted NMR parameter. Apparent self-diffusion, D_app_, is one experimental descriptor used to interrogate translational mobility and restriction relevant to accessibility, but D_app_ and accessibility are not synonymous. Accessibility additionally reflects the combined effects of architecture, connectivity, tortuosity, compartment dimensions, interfaces, exchange and the measurement scale.

Accessibility is therefore not synonymous with moisture content, water activity, water-holding capacity, expressible water, “free water” or a particular T_2_ component. Rather, accessibility is a continuum determined by structural resistance to molecular displacement. Relevant architectural features include network density, pore geometry, connectivity, tortuosity, interfaces, compartment dimensions and exchange.

This yields a central principle: retained water is not necessarily accessible water. A matrix may retain substantial amounts of water during storage or cooking while strongly limiting translational redistribution. Table 1 summarizes the preferred interpretation and principal cautions for the major TD-NMR descriptors used in food systems.

## 9. A T_2_–D Molecular-State Map

Considering relaxation and diffusion jointly provides a useful representation of food-water state. Four limiting regimes can be considered: high T_2_–high D, low T_2_–high D, high T_2_–low D, and low T_2_–low D. The high T_2_–low D regime is particularly instructive because it shows that relatively mobile local water may nevertheless experience restricted longer-range displacement.

This two-dimensional representation moves interpretation beyond a simple bound/free classification toward a more physically meaningful combination of local molecular state and mesoscale structural restriction. The conceptual T_2_–D state space is illustrated in Figure 1.

## 10. Multidimensional TD-NMR and Exchange

Two-dimensional T_1_–T_2_ measurements can resolve correlated proton populations that overlap in one-dimensional relaxation measurements. D–T_2_ measurements are particularly relevant because they combine local relaxation information with translational displacement. Hürlimann et al. demonstrated quantitative characterization of food products using two-dimensional D–T_2_ and T_1_–T_2_ distribution functions [24].

Exchange-sensitive measurements add a further structural dimension by probing whether distinct molecular environments communicate. A food can contain multiple hydrated domains yet exhibit restricted macroscopic transport if those domains are poorly connected. Future interpretation should therefore increasingly consider molecular state → compartmentalization → connectivity → accessibility.

## 11. Meat and Muscle Foods: The Classical Model

Evidence boundary for application examples: Section 11, Section 12, Section 13, Section 14, Section 15, Section 16 and Section 17 provide representative mechanistic examples rather than a systematic quantitative synthesis. Reported associations should therefore not be interpreted as universal effect sizes across food matrices. Direct comparison among studies is constrained by differences in composition, sample size, biological replication, field strength, temperature, pulse sequence, inversion/fitting procedures and statistical analysis. Where evidence is limited to particular formulations or experimental conditions, the interpretation should remain system-specific.

Meat remains one of the best-established food applications of low-field NMR. Muscle water is organized within a hierarchical architecture involving myofibrils, sarcomeres, connective tissue and extracellular spaces. Postmortem biochemical changes, pH, contraction and heating alter this architecture and redistribute water.

Bertram et al. demonstrated a strong relationship between meat microstructure and T_2_ relaxation [16]. Subsequent studies showed that cooking-induced changes in protein structure produce systematic changes in water mobility and distribution [17]. Combined microscopy and LF-NMR further demonstrated relationships among structural evolution, water distribution, water-holding capacity and cooking loss [18], while Pearce et al. reviewed water distribution and mobility during the conversion of muscle to meat [19].

Self-diffusion adds another level by probing molecular displacement through that architecture. Laghi et al. demonstrated the ability of water diffusion to assess meat microstructure [23], and recent relaxometry work has extended TD-NMR toward technological classification of pork hams [6].

## 12. Plant-Based Meat Analogues: A New Structural Challenge

Plant-based meat analogues are especially important because their internal architecture is engineered rather than inherited from native muscle. These products combine proteins, polysaccharides, lipids and water through extrusion, shear processing, gelation, emulsification or related structuring approaches [25,26].

Recent TD-NMR studies demonstrate clear differences between animal- and plant-based burger architectures. Wiesman et al. showed that TD-NMR relaxation and two-dimensional T_1_–T_2_ fingerprints could distinguish the molecular organization of beef and soy-based meat-analogue burgers and relate these differences to microscopy and water behavior [7]. Azachi and Wiesman subsequently integrated relaxation, self-diffusion, texture profile analysis and Cryo-SEM [8].

The importance of intrinsic proton mobility to plant-based product texture was independently supported by Arildsen Jakobsen et al., who found that changing the ratio of high- and low-moisture texturized vegetable proteins altered LF-NMR T_2_ behavior together with sensory texture [9]. These studies support a shift from viewing water merely as a formulation component toward considering hydration architecture.

## 13. Dairy Systems, Gels and Emulsions

Dairy products, protein gels and food emulsions provide particularly informative model systems for relating molecular water mobility to evolving food architecture. Their interconnected aqueous, protein, lipid and polysaccharide phases change during gelation, acidification, ripening, syneresis, storage and destabilization. LF-NMR studies of acidified milk systems have demonstrated that changes in relaxation behavior can be related to protein composition, microstructural organization and the appearance of expelled serum during syneresis [27,28]. Protein formulation can likewise modify network density, texture, water-holding behavior and the relaxation characteristics of heat- and acid-induced milk gels [29].

These systems illustrate why multimodal characterization is important. Relaxation measurements report the molecular consequences of network formation and water–matrix interactions, whereas microscopy and rheology provide complementary information on morphology and bulk mechanical behavior. Recent work also demonstrates the ability of LF-NMR to characterize aqueous- and lipid-phase mobility and detect formulation-, processing- and storage-induced destabilization in emulsions [30]. Thus, changes in T_1_ or T_2_ should preferably be interpreted together with independent structural or mechanical measurements rather than assigned uniquely to a particular water population.

## 14. Cereals and Starch-Rich Foods

Starch-rich foods undergo extensive water redistribution during mixing, gelatinization, baking, cooling and storage. A comprehensive review by Riley et al. described the use of time-domain ^1^H NMR for studying proton dynamics in starch-rich foods [31].

Bread staling provides a particularly clear example of water as an endogenous structural reporter. Bou Orm et al. showed that heating rate during baking altered proton T_2_ relaxation together with starch microstructure and mechanical properties during subsequent staling [32]. Thus, water is not only an ingredient being quantified; it is also a probe of structural evolution.

## 15. Freezing and Frozen Foods

Freezing illustrates clearly why water quantity cannot describe water function. Ice formation changes the physical state and distribution of water without necessarily changing total water content. TD-NMR is sensitive to these transformations because highly mobile and rigid proton populations have very different relaxation behavior.

LF-NMR is also well suited to monitoring redistribution among proton environments during frozen storage and freeze–thaw cycling. Such measurements can reveal changes in the relative populations and mobility of water associated with ice formation, recrystallization, concentration of the unfrozen phase and progressive disruption of protein or polysaccharide networks. These molecular-state measurements are particularly informative when combined with MRI, calorimetry, rheology or microscopy because changes in relaxation alone should not be interpreted as a unique measure of ice-crystal structure.

A major recent development is the combination of LF-NMR with predictive algorithms. Jiang et al. used transverse-relaxation measurements together with partial least-squares regression and a back-propagation artificial neural network to assess frozen model foods non-destructively [10]. This progression can be represented as TD-NMR signal → molecular fingerprint → predictive model → quality assessment.

## 16. Drying and Rehydration

Drying is fundamentally a coupled heat- and mass-transport process. The rate of moisture removal depends not only on how much water remains but also on its molecular state and on the pathways available for migration from internal regions toward an evaporating surface. LF-NMR has therefore been increasingly used to follow water redistribution during drying. Early work demonstrated its application to beef granules [33], while more recent studies have followed water mobility and distribution during alternative drying regimes in plant-derived foods [34,35].

The accessibility framework suggests an important extension: relaxation identifies how the molecular environments of remaining water evolve, while diffusion can potentially determine whether that remaining water retains significant translational mobility. This distinguishes water remaining in the matrix from water capable of moving through the matrix.

The same reasoning applies in reverse during rehydration. Dynamic LF-NMR measurements can distinguish bulk water uptake from progressive immobilization of water within an evolving hydrated structure. Recent measurements on texturized soy protein demonstrated that rehydration kinetics and intrinsic water mobility can be followed directly through changes in T_2_, providing an example of TD-NMR as a time-resolved probe of structural hydration rather than merely an endpoint moisture measurement [36].

## 17. Lipids, Edible Oils and Oxidation

TD-NMR is not limited to aqueous food systems. Lipids provide strong ^1^H signals, and relaxation and diffusion respond to molecular organization, viscosity, crystallization and chemical transformation [37,38].

Osheter et al. demonstrated that LF-NMR self-diffusion changes systematically during thermal oxidation and can correlate with conventional oxidation indices [11]. The same analytical platform was subsequently combined with artificial-intelligence classification to predict oil oxidation state [12]. These studies illustrate that TD-NMR need not measure a conventional chemical marker directly if the chemical transformation produces a reproducible molecular-dynamic fingerprint.

## 18. Translating Molecular Descriptors into Food Function

The greatest value of TD-NMR emerges when molecular-state measurements can be connected to macroscopic food performance. Texture is determined by composition, network geometry, phase organization, hydration and deformation; these same features influence relaxation and diffusion. Associations between TD-NMR descriptors and texture are therefore physically plausible, but a particular T_2_ value cannot be assumed to predict hardness across unrelated food matrices.

Repeated TD-NMR measurements can also provide molecular fingerprints of product ageing, cooking, pressing, freezing and drying, potentially supporting predictive shelf-life and process models.

## 19. Juiciness: A Model Case for Water Accessibility

Juiciness provides perhaps the clearest demonstration of the distinction between water quantity and water function. For retained water to contribute to juiciness, fluid must become available during biting and mastication. Thus, high moisture content does not necessarily imply high perceived juiciness.

A proposed mechanistic sequence is food architecture → water compartmentalization and pathway connectivity → translational accessibility → fluid/serum release during mastication → oral lubrication → perceived juiciness. Classic meat studies established relationships among water holding, cooking loss and juiciness [39]. More recently, Zhang et al. showed that the temporal development of juiciness in beef and plant-based patties was strongly related to serum release during the early stages of mastication [40].

An important distinction must be retained: NMR-derived water accessibility is a physicochemical construct, whereas mechanically induced serum release and juiciness are different functional outcomes. Direct experimental evidence establishing that an NMR-derived accessibility descriptor predicts serum release during mastication and subsequent sensory juiciness within the same independent samples remains limited. Current evidence supports separate links in the proposed chain: TD-NMR can characterize molecular mobility and restriction, while serum release during early mastication has been associated with temporal juiciness perception [40]. These observations motivate, but do not yet establish, the complete causal sequence D_app_ → serum release → oral lubrication → perceived juiciness. Validation requires matched TD-NMR, structural, mechanical-release, oral-processing and sensory measurements performed on appropriately matched experimental units (Table 2).

## 20. Multimodal Validation and Complementary Measurements

TD-NMR measures molecular relaxation and translational displacement but cannot uniquely identify the geometry or thermodynamic state producing a particular NMR signature. Complementary measurements are therefore essential for mechanistic validation. MRI provides spatially resolved proton distributions; DSC probes thermal transitions and, under defined assumptions, freezable fractions; NIR provides composition-sensitive spectral fingerprints; water activity characterizes thermodynamic availability; water-holding capacity and expressible-water tests quantify perturbation-dependent retention or release; microscopy and X-ray micro-CT describe architecture; and rheology or texture analysis quantify bulk mechanical behavior. These methods should not necessarily converge numerically because they interrogate different physical properties and length/time scales. Agreement can strengthen a structural interpretation, whereas systematic divergence may itself reveal mechanistically important differences between thermodynamic availability, molecular mobility, spatial organization, mechanical retention and transport.

## 21. Chemometrics, Machine Learning and Artificial Intelligence

Modern TD-NMR experiments can generate far richer datasets than a single relaxation time, including complete T_1_ and T_2_ distributions, diffusion attenuation curves, apparent D, T_1_–T_2_ maps, D–T_2_ maps, exchange descriptors and repeated measurements during processing.

The recent frozen-food study of Jiang et al. [10] and the LF-NMR oil-oxidation classifier [12] demonstrate two different applications: prediction of quality attributes and classification of degradation state. Future progress should move beyond purely black-box classification by preserving a physically interpretable hierarchy: raw TD-NMR signal → relaxation/diffusion descriptors → molecular-state representation → structural/function model → quality prediction.

For predictive use, internal cross-validation alone is insufficient evidence of generalizability. Robust models should, where feasible, be evaluated on independent batches and external samples and challenged for transfer across instruments, operators and acquisition dates. Particular attention is required on small-sample/high-dimensional overfitting, preprocessing leakage, batch effects, calibration drift and domain shift. Model reporting should include training/validation/test separation, replicate structure, preprocessing fitted only within training data, uncertainty or confidence estimates, and interpretable feature attribution or other evidence that predictions depend on physically meaningful TD-NMR information rather than incidental batch-specific signals.

## 22. Non-Invasive and Industrial TD-NMR

The practical properties of TD-NMR are attractive for industrial quality assessment. Measurements can be rapid, reagent-free, non-destructive and compatible with automation. Portable, sensor-oriented and industrial applications have been reviewed extensively [13,14,15,41,42], although challenges remain in calibration transfer, sample geometry, temperature control and integration into manufacturing environments.

An especially important recent development is direct analysis of packaged food. Almeida et al. showed that radiofrequency transparency of many packaging materials can enable relaxometry and diffusion measurements without opening the product [5]. Potential applications include incoming-ingredient control, formulation verification, batch comparison, oxidation assessment, storage monitoring and packaged-product evaluation.

This capability is conditional rather than universal. Metallic packaging and strongly RF-attenuating or multilayer barrier structures may prevent or distort measurement; package geometry and filling factor can alter sensitivity; product temperature must be controlled because relaxation and diffusion are temperature-dependent; heterogeneous packages may create sampling-representativeness limitations; and industrial deployment requires procedures for instrument-drift monitoring, reference standards and calibration transfer across instruments, batches and packaging formats.

## 23. TD-NMR as a Process Analytical and Molecular-State Sensing Platform

Food-manufacturing processes are conventionally monitored through variables such as temperature, pressure, shear, residence time and formulation. These parameters describe what has been applied to the food but do not necessarily quantify the material state actually produced.

TD-NMR offers a route to measuring part of this hidden internal state. A future process architecture may therefore take the form composition + processing → food architecture → TD-NMR measurement of molecular state → quality/function prediction → process intervention.

Digital manufacturing requires models capable of estimating the evolving state of a material rather than merely recording its process history. TD-NMR is attractive in this context because relaxation and diffusion respond directly to hydration, molecular restriction, phase organization and transport.

A future architecture-aware workflow may consist of real food → TD-NMR + complementary sensors → molecular/structural state estimation → physics-based and machine-learning models → predicted quality and function → manufacturing intervention → updated measurement. TD-NMR would contribute a molecular-dynamic layer that is often absent from conventional manufacturing sensors.

Although most current food applications remain at the level of off-line or at-line characterization, these developments establish the analytical basis for a future process-analytical role of TD-NMR. Such a transition will require robust calibration transfer, rapid acquisition, standardized sample presentation and prospective validation under manufacturing conditions. Accordingly, closed-loop TD-NMR-guided manufacturing should presently be regarded as an emerging opportunity rather than an established industrial capability.

## 24. Standardization and Reproducibility

The growing sophistication of TD-NMR makes standardized reporting increasingly important. Relaxation measurements depend on magnetic-field strength or proton frequency, sample temperature and geometry, pulse calibration, repetition time, echo spacing, number of echoes/scans, signal-to-noise ratio, fitting methodology and, where distributions are reconstructed, inverse Laplace regularization and peak-selection criteria.

Diffusion measurements additionally depend on gradient calibration, gradient strength, gradient duration, diffusion interval, pulse-sequence design, sample temperature and diffusion model. Reporting only a fitted T_2_ or D value without these acquisition conditions greatly reduces interlaboratory comparability. Predictive models should also disclose training/test separation, validation procedures, preprocessing and model-transfer procedures.

## 25. Limitations and Interpretative Boundaries

TD-NMR provides powerful molecular information, but relaxation components do not always correspond uniquely to specific chemical or spatial water populations; exchange can shift, merge or broaden apparent populations; water and lipid proton signals may overlap; relaxation and diffusion are strongly temperature-dependent; inverse Laplace transformation is ill-conditioned; and apparent diffusion depends on observation time and geometry.

Empirical correlations between NMR parameters and food quality do not by themselves establish causation. Statements such as “T_2_ equals water binding” or “D directly measures pore connectivity” should therefore be avoided. A more rigorous formulation is that T_1_, T_2_ and D are experimentally defined molecular descriptors whose interpretation depends on composition, exchange, architecture, temperature and measurement scale. The principal analytical strengths, limitations and recommended safeguards are summarized in Table 3. The minimum recommended reporting information for quantitative and reproducible food TD-NMR studies is summarized in Table 4.

## 26. A Practical Hierarchy for Selecting TD-NMR Measurements

Not every application requires every TD-NMR technique. A useful hierarchy is: Level 1—quantity (^1^H signal amplitude or conventional moisture analysis); Level 2—molecular state (T_1_/T_2_); Level 3—molecular heterogeneity (relaxation distributions); Level 4—translational accessibility (self-diffusion D); Level 5—correlated molecular state (T_1_–T_2_ or D–T_2_); Level 6—connectivity and dynamic evolution (exchange-sensitive or time-resolved measurements).

The measurement strategy should be determined by the physical question rather than by the availability of increasingly complex pulse sequences.

## 27. From Molecular-State Measurement to Predictive Food Design

The concepts developed throughout this review can be summarized as a proposed mechanistic framework: composition + processing → food architecture → water quantity and molecular state → local mobility and compartmentalization/connectivity → translational accessibility → transport/release → food function.

The central advantage of TD-NMR is its ability to interrogate several intermediate levels that are poorly represented by conventional compositional measurements. Moisture analysis measures the amount of water; relaxation describes how molecular environments affect spin dynamics; diffusion introduces the ability of molecules to translate through the surrounding architecture; and exchange measurements can further describe communication between compartments. Figure 2 summarizes the resulting architecture–mobility–accessibility–function framework.

## 28. Future Research Priorities and Predictive Food Design

Future research should move away from universal assignments of bound, immobilized and free water unless explicitly defined; distinguish the physical concept of accessibility from the experimental descriptor D_app; combine relaxation and diffusion when transport, migration or release are central; explore multiple diffusion times when structural restriction is interpreted; integrate NMR descriptors with porosity, connectivity, tortuosity, interfaces and mechanics; validate TD-NMR against complementary thermodynamic, structural and functional measurements; increase dynamic measurements during processing; standardize protocols, uncertainty reporting and reference materials; preserve physically meaningful descriptors in machine-learning systems and require independent validation; validate accessibility–serum-release–sensory relationships using matched experimental units; and test whether molecular-state descriptors can serve as real-time manufacturing control variables.

Traditional analytical food science asks what properties the product possesses. Predictive food engineering asks what internal state must be generated to produce a desired function. From this perspective, moisture is only the first layer.

If function depends on water movement, the design objective must include an appropriate transport architecture. For juiciness, this may mean retaining sufficient water during manufacturing and storage while permitting controlled fluid mobilization during mastication. For drying, transport pathways must remain effective without excessive structural collapse. For freezing, the distribution and mobility of unfrozen water must be controlled while minimizing destructive ice-associated structural change.

The design hierarchy becomes target function → required transport behavior → required molecular/architectural state → ingredient composition + processing trajectory. TD-NMR contributes by making important elements of that otherwise hidden internal state experimentally observable.

## 29. Conclusions

Moisture content remains indispensable to food analysis, but the amount of water alone cannot fully describe how water contributes to food behavior. Function depends on interactions among water, macromolecules, interfaces, compartments and transport pathways generated by food architecture.

TD-NMR provides a useful analytical window into these properties. T_1_ and T_2_ characterize relaxation environments and molecular state; relaxation distributions describe heterogeneity; self-diffusion adds a transport-sensitive measure of translational displacement; and multidimensional and exchange-sensitive experiments can further characterize correlated molecular states and communication between compartments.

The central progression proposed in this review is water quantity → molecular state → local mobility and compartmentalization → translational accessibility → transport/release → food function. The next generation of food TD-NMR should move beyond empirical relaxation fingerprints toward quantitative structure–transport–function relationships, standardized protocols, multimodal sensing and physically informed machine learning. In this role, TD-NMR can evolve from a rapid laboratory method for composition and relaxation analysis into a molecular-state sensing platform for predictive food quality, product design and intelligent manufacturing.

## Figures and Tables

**Figure 1:**
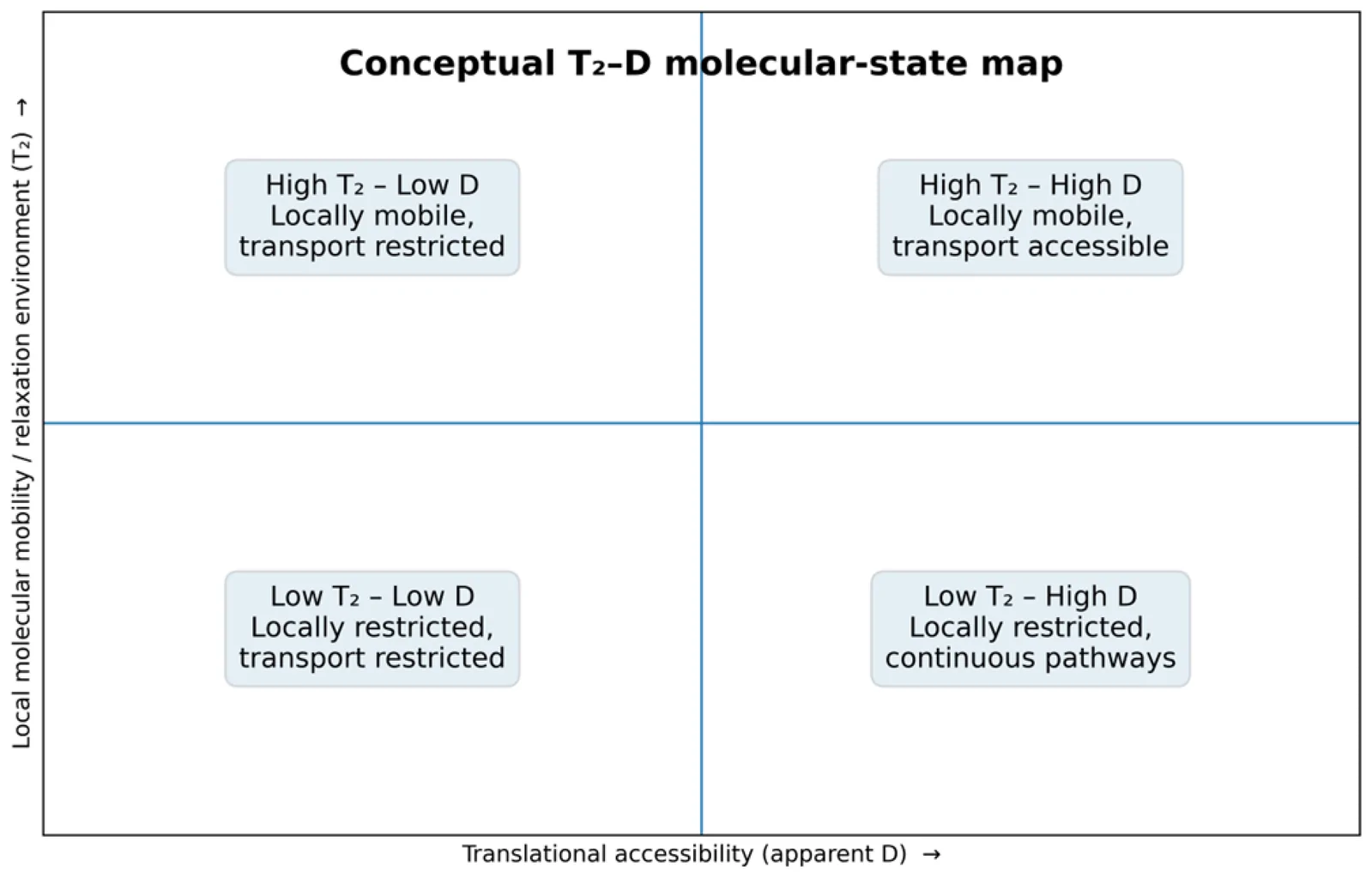
Conceptual T_2_–D molecular-state map for food water. The vertical dimension represents the local relaxation environment reflected by T_2_, whereas the horizontal dimension represents translational accessibility reflected by apparent self-diffusion, D. The four limiting regimes illustrate why local molecular mobility and longer-range accessibility are not equivalent.

**Figure 2:**
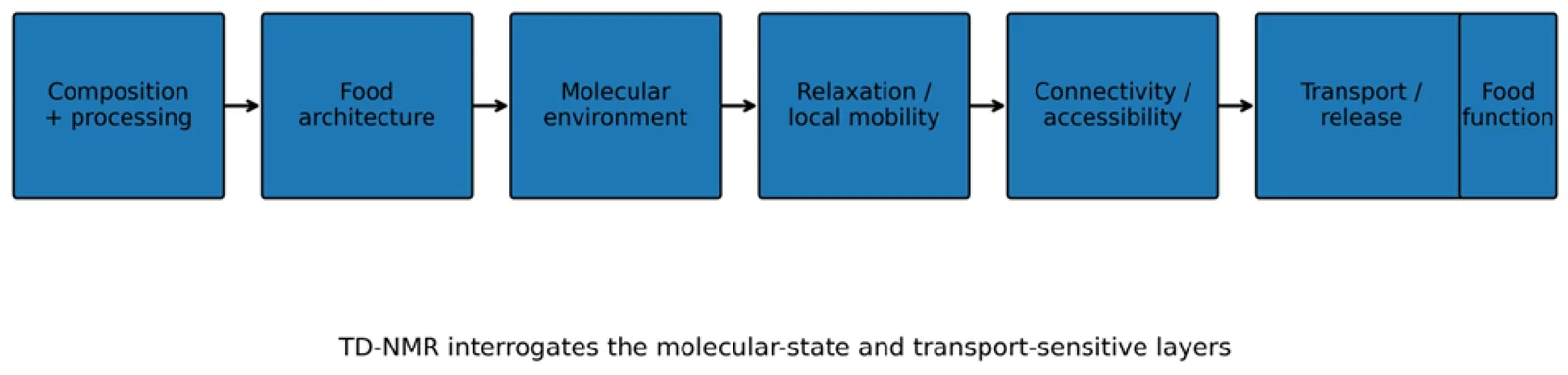
Architecture–mobility–accessibility–function framework. Composition and processing generate food architecture, which governs molecular environment, relaxation behavior, connectivity and translational displacement. TD-NMR probes molecular-state and transport-sensitive layers that contribute to macroscopic food function.

**Table 1:** Major TD-NMR descriptors and their preferred interpretation in food systems.

TD-NMR Descriptor	Primary Sensitivity	Preferred Interpretation	Important Caution
^1^H signal amplitude	Proton abundance	Water/lipid quantity after calibration	Does not describe molecular state
T_1_	Longitudinal relaxation dynamics	Molecular environment and mobility	Field- and temperature-dependent
T_2_	Transverse relaxation dynamics	Molecular restriction and local environment	Not a direct transport measurement
T_2_ distribution	Heterogeneity of relaxation environments	Molecular-state fingerprint	Populations are not necessarily discrete water species
D_app	Translational displacement	Experimental descriptor of translational mobility and restriction relevant to accessibility	Depends on diffusion time, geometry, exchange and acquisition conditions
T_1_–T_2_	Correlated relaxation	Separation of overlapping proton environments	Inverse problem and acquisition complexity
D–T_2_	Diffusion + relaxation	Coupled relaxation–translational behavior	Requires careful physical interpretation
Exchange-sensitive NMR	Transfer among environments	Compartment communication/connectivity	Model- and timescale-dependent

**Table 2:** Representative food applications interpreted through the quantity–state–accessibility–function framework.

Food System	Principal TD-NMR Descriptors	Molecular/Structural State Represented	Accessibility/Transport Interpretation	Relevant Food Function
Meat	T_2_ distributions, D_app_, MRI	Water compartmentalization, redistribution and restriction within muscle structure	Mobility and connectivity of aqueous domains; potential for redistribution and release	WHC, cooking loss, tenderness, juiciness
Plant-based meat	T_1_, T_2_, D_app_, 2D maps	Hydration state and water compartmentalization within protein–polysaccharide networks	Water accessibility, network connectivity and transport restriction	Texture, structural mimicry, water retention, juiciness
Dairy products and gels	T_1_, T_2_	Water redistribution and restriction during network formation	Availability and mobility of water within evolving gel networks	Gelation, syneresis, texture
Cereal products	T_2_ distributions	Water redistribution associated with starch and protein transformations	Mobility and redistribution among cereal components and domains	Baking performance, staling, softness
Frozen foods	T_2_ distributions, multivariate/ML analysis	Freezing- and thawing-induced changes in molecular mobility and compartmentalization	Redistribution and altered accessibility of unfrozen/mobile water	Drip loss, texture, freeze–thaw stability
Dried foods	T_1_, T_2_, potentially D_app_	Residual water populations with different degrees of molecular restriction	Distinction between remaining water and water capable of redistribution or transport	Drying endpoint, storage stability, rehydration
Edible oils	T_1_, T_2_, D	Changes in molecular mobility and relaxation associated with oxidation and compositional evolution	Molecular mobility as an indicator of evolving physicochemical state rather than water accessibility	Oxidative stability and quality
Packaged foods	Relaxometry, diffusion	Non-invasive assessment of compositional and molecular/structural state	Detection of changes in mobility, compartmentalization or transport without destructive sampling	Quality control, authenticity, spoilage detection

**Table 3:** Strengths, limitations and recommended safeguards for TD-NMR food analysis.

Strength	Analytical Opportunity	Main Limitation	Recommended Safeguard
Non-destructive measurement	Repeated monitoring	Signal assignments may be ambiguous	Use complementary structural measurements
Rapid relaxation analysis	Routine QC	Acquisition conditions affect values	Standardize temperature and pulse parameters
Relaxation distributions	Detect heterogeneous states	Ill-conditioned inversion	Report SNR, regularization, inversion and peak-selection details
Self-diffusion	Transport-sensitive descriptor	Observation-scale dependence	Report δ, Δ, gradient range/calibration and temperature; use multiple Δ when restriction is inferred
2D correlation	Resolves overlapping populations	Greater acquisition/analysis complexity	Use physically constrained interpretation
Minimal preparation	Preserves architecture	Geometry may influence measurement	Standardize sample geometry and filling factor
High-dimensional fingerprints	ML/AI prediction	Risk of overfitting and domain shift	Independent validation, transparent preprocessing and transfer testing
Compact instrumentation	Industrial deployment	Calibration transfer and instrument drift	Reference standards, drift monitoring and transfer protocols

**Table 4:** Minimum recommended reporting information for quantitative and reproducible food TD-NMR studies.

Category	Minimum Information to Report	Purpose
Sample and environment	Food formulation/state; biological and technical replicate structure; sample geometry/filling; temperature and equilibration procedure	Defines the measured material state and supports repeatability
Instrument and calibration	Instrument/model; magnetic-field strength or ^1^H frequency; probe/sample dimensions; pulse and receiver calibration; relevant reference standards	Supports interlaboratory and longitudinal comparability
Relaxation acquisition	Sequence; repetition/recovery time; echo spacing; number of echoes; scans/averages; acquisition window; SNR or noise estimate	Allows assessment of relaxation bias and data quality
Distribution/ILT analysis	Inversion algorithm; regularization method and parameter selection; discretization/range; baseline/noise treatment; peak identification/number-selection rule; replicate uncertainty	Limits over-interpretation of ill-posed inverse solutions
Diffusion	Sequence; gradient calibration/range; gradient duration δ; diffusion interval Δ; temperature; attenuation model; D_app_ uncertainty; multiple Δ values when restriction/accessibility is inferred	Defines the observation window and scale dependence of D_app
Statistics and uncertainty	Independent sample n; technical repeats; aggregation rule; uncertainty/CI; statistical tests and assumptions; effect estimates where appropriate	Separates analytical repeatability from biological/product variability
Predictive models/ML	Feature preprocessing; train/validation/test separation; batch structure; external validation; cross-instrument/batch transfer where relevant; uncertainty; interpretability/feature-attribution method	Reduces leakage and overfitting and tests practical generalizability

## Data Availability

No new data were created or analyzed in this study. Data sharing is not applicable to this article.
